# Genetic propensity, socioeconomic status, and trajectories of depression over a course of 14 years in older adults

**DOI:** 10.1038/s41398-023-02367-9

**Published:** 2023-02-23

**Authors:** Martyna Kosciuszko, Andrew Steptoe, Olesya Ajnakina

**Affiliations:** 1 Faculty of Biology, Medicine and Health, Division of Developmental Biology and Medicine, University of Manchester, Manchester, UK; 2 Department of Social Statistics, The Cathie Marsh Institute, University of Manchester, Manchester, UK; 3 Department of Behavioural Science and Health, Institute of Epidemiology and Health Care, University College London, London, UK; 4 Department of Biostatistics & Health Informatics, Institute of Psychiatry, Psychology and Neuroscience, King’s College London, London, UK

**Keywords:** Depression, Predictive markers, Personalized medicine, Clinical genetics

## Abstract

Depression is one of the leading causes of disability worldwide and is a major contributor to the global burden of disease among older adults. The study aimed to investigate the interplay between socio-economic markers (education and financial resources) and polygenic predisposition influencing individual differences in depressive symptoms and their change over time in older adults, which is of central relevance for preventative strategies. The sample encompassing *n* = 6202 adults aged ≥50 years old with a follow-up period of 14 years was utilised from the English Longitudinal Study of Ageing. Polygenic scores for depressive symptoms were calculated using summary statistics for (1) single-trait depressive symptoms (PGS-DS_single_), and (2) multi-trait including depressive symptoms, subjective well-being, neuroticism, loneliness, and self-rated health (PGS-DS_multi-trait_). The depressive symptoms over the past week were measured using the eight-item Centre for Epidemiologic Studies Depression Scale. One standard deviation increase in each PGS was associated with a higher baseline score in depressive symptoms. Each additional year of completed schooling was associated with lower baseline depression symptoms (*β* = −0.06, 95%CI = −0.07 to −0.05, *p* < 0.001); intermediate and lower wealth were associated with a higher baseline score in depressive symptoms. Although there was a weak interaction effect between PGS-DSs and socio-economic status in association with the baseline depressive symptoms, there were no significant relationships of PGS-DSs, socio-economic factors, and rate of change in the depressive symptoms during the 14-year follow-up period. Common genetic variants for depressive symptoms are associated with a greater number of depressive symptoms onset but not with their rate of change in the following 14 years. Lower socio-economic status is an important factor influencing individual levels of depressive symptoms, independently from polygenic predisposition to depressive symptoms.

## Introduction

Depression is one of the leading causes of disability worldwide contributor to the global burden of disease [1]. It is also one of the most common geriatric psychiatric disorders [2] with the prevalence of depression increasing as people age and peaking in adults aged between 55 and 74 years old [3]. Considering the rapid population ageing is a worldwide phenomenon, there is a pressing need to have a better understanding of underlying depression risk in older adults.

Depression has a complex and multifactorial aetiology, which is comprised of both environmental and genetic factors [4, 5]. Indeed, increasing evidence suggests that in older adults, socio-economic status, as measured by educational attainment and wealth, is significantly associated with depression onset [6–9]. Educational attainment represents a socio-economic position achieved during the first decades of life; whereas wealth reflects a socio-economic status obtained towards later stages of life and is likely to reflect opportunities, or lack of them, throughout the adult life [10]. Therefore, it was important to model these socio-economic factors separately to disentangle their effects in relation to depression onset and its trajectory in older adults [10].

Depression is also a heritable disorder, with adult twin studies having estimated its average heritability between 30–50%, which further increases linearly with age [11, 12]. More recent genome-wide association studies (GWASs) have revealed that the genetic architecture of the broad phenotype of depression (meaning it included clinically diagnosed depression, self-reported diagnosis of depression and/or depressive symptoms as part of the definition), is characterised by multiple common genetic markers spread across the entire genome [13–15]. Building on the results from GWASs, polygenic score (PGS), which measures an individual genetic propensity to a trait by combining the effects of many common genetic variants associated with it [16], confirmed that depressive symptoms are polygenic in nature [17]. PGS for depressive symptoms has further been shown to be a good indicator of an individual’s genetic liability to develop depressive symptoms in both clinical and population cohorts [18]. However, it is not known if PGSs for depressive symptoms also influence the rate of change in depressive symptoms over time in older adults.

PGSs can further be used to assess predisposition to a condition that may never be expressed phenotypically, highlighting a shared genetic risk between traits and health conditions [19, 20]. Indeed, PGS analyses revealed a shared polygenic contribution between depression diagnosis and schizophrenia, bipolar disorders, attention deficit hyperactivity disorder, autism, migraines, body mass index and Alzheimer’s disease [21] as well as cognitive ability [22] and educational attainment [14]. It, therefore, may be argued that to fully capture the impact of polygenic influences on depressive symptoms, the genetic information from the traits that correlate with depressive symptoms may need to be incorporated into PGS [23]. This in turn may provide more detailed insights into the genetic make-up of depressive symptoms, and their trajectories, informing the search for their biological mechanisms.

Because genetic variants are segregated at conception and determined randomly, it may be assumed that genetic predisposition to depressive symptoms is deterministic. However, it is equally possible that a higher polygenic predisposition may exacerbate the effect of lower educational attainment and wealth in moderating the risk of depressive symptoms over time. A clearer understanding of this gene-by-environment interaction (GxE) will help understand how polygenic predisposition to depressive symptoms interacts with these socio-economic factors in influencing the risk of depressive symptoms onset and their longitudinal trajectory in older adults. This knowledge is essential if clinical decisions should be guided by risk models, especially those that include PGSs [24].

Therefore, drawing on a large, phenotypically well-defined sample of older adults, we investigated if a higher polygenic predisposition to depressive symptoms was associated with individual differences in depressive symptoms at baseline and a rate of change in depressive symptoms across five time points measured sequentially over the 14-year follow-up. We further tested the interactions between PGS for depressive symptoms with educational attainment and wealth in relation to onset and rate of change in depressive symptoms during the follow-up. We also investigated if utilising a measure of polygenic predisposition to depressive symptoms based on multiple correlated traits provides a stronger predictor of depressive symptoms onset and their longitudinal trajectory over 14 years than a single trait PGS.

## Methods

### Study participants

We used data from the English Longitudinal Study of Ageing (ELSA), which is an ongoing large, multidisciplinary study of the English population aged ≥50 years [25]. The ELSA study started in 2002-2003 (wave 1) with participants recruited from the Health Survey for England, which was designed to monitor the health of the general population, who were then followed up every 2 years. The ELSA sample is periodically refreshed with younger participants to ensure that the full age spectrum is maintained [25]. Compared with the national census, the ELSA sample has been shown to be representative of the non-institutionalised general population aged ≥50 residing in the UK [25]. Because the blood samples (for genetic data) were collected by nurses during a home visit at wave 2 (2004-2005) for the core members who started at wave 1 and wave 4 (2008-2009) for the participants joining the study at wave 4 through the refreshment sample, the data from these waves formed our baseline. Participants who reported a history of schizophrenia, psychosis, bipolar disorder, and anxiety disorders at the baseline and during follow-up were excluded from the analysis. Each of the ELSA waves received ethical approval from the National Research Ethics Service (London Multicentre Research Ethics Committee) and all participants gave informed consent.

### Study variables

#### Depressive symptoms

At baseline and at each wave of data collection following the baseline (i.e., wave 5—2010/11, wave 6—2012/13, wave 7—2014/15, wave 8—2016/17, and wave 9—2018/19), the self-reported depressive symptoms over the past week were measured using the eight-item Centre for Epidemiologic Studies Depression Scale (CES-D) [26]. Each CES-D item was scored on a binary point response scale (anchored at 1 = ‘*yes*’; 0 = ‘*no*’). Scores were summed to generate a total continuous score of depressive symptoms, ranging from 0 (‘*no depressive symptoms*’) to 8 (‘*severe depressive symptoms*’) (Supplementary Table 2). The Cronbach’s alpha in this sample was 0.80. CES-D measure is a well-recognised measure of depressive symptoms in population-based studies [27, 28].

#### Socio-economic indicators

Educational attainment at baseline was measured as the number of years of completed schooling. Wealth at baseline was calculated by summing wealth from property, possessions, housing, investments, savings, artwork, jewellery, and net of debt [25]. Because incomes among older people often do not reflect well the available financial resources, it was not included in the analyses [25]. To provide more insight into the effects of different levels of wealth, this variable was divided into high, intermediate, and low levels using the interquartile range.

#### Covariates

The covariates include sex and age; to capture the non-linear effects of ageing, we further included age^2^ as a covariate. Genetic ancestry (as was measured with 10 principal components (see below)), was included among the covariates to account for any ancestry differences in genetic structures that could bias our results [29]. We have further tested for the interaction effect between polygenic scores for depression and sex, and the interaction between age and sex. As these interactions were not significant, they were not included in the models.

### Genetic data

The genetic data were extracted from the blood draws taken during home visits. The genome-wide genotyping was performed at University College London Genomics in 2013–2014 using the Illumina HumanOmni2.5 BeadChips (HumanOmni2.5-4v1, HumanOmni2.5-8v1.3), which measures ~2.5 million markers that capture the genomic variation down to 2.5% minor allele frequency (MAF).

#### Quality control

In the ELSA cohort, 9542 participants gave a blood sample; of these, 7189 (75.45%) passed the quality control post genome-wide genotyping. The methods employed for quality control of genomic data in the ELSA study are those outlined by the Health and Retirement Study [30] This was done to harmonise the research across the age-related longitudinal studies by adopting a consistent methodology. Single-nucleotide polymorphism (SNPs) were excluded if they were non-autosomal, MAF was <1%, if more than 2% of genotype data were missing and if the Hardy-Weinberg Equilibrium *p* < 10^−4^. Samples were removed based on call rate (<0.99), heterozygosity, relatedness and if the recorded sex phenotype was inconsistent with genetic sex. To identify ancestrally homogenous analytic samples the ELSA genomic samples use a combination of both self-reported ethnicity and analyses of genetic ancestry. Genetic ancestry was estimated via comparison of participants’ genotypes to global reference populations using principal component analyses (PCA) [30, 31]. Because PCA allows examining population structure in a cohort by determining the average genome-wide genetic similarities of individual samples, derived principal components (PCs) can be used to group individuals with shared genetic ancestry, to identify outliers, and as covariates, to reduce false positives due to population stratification [32]. Although up to 98% of the ELSA participants self-described to be of European cultural background, PC highlighted the present of ancestral admixture in *n* = 65 (0.9%) individuals (implying these individuals had ancestors from two or more populations [32]. Even though this type of labelling of ancestral populations oversimplifies the complexity of human genetic variation, accounting for systematic differences in allele frequencies is necessary for genetic analyses [32]. Therefore, these participants with ancestral admixture were removed from the analyses. The final sample includes all self-reported European participants that had PC loadings within ± one standard deviations of the mean for eigenvectors one. PCs were then re-calculated to further account for population stratification. Therefore, our analytic sample included the full ELSA sample that provided genetic samples and passed quality control. We further utilized the PCs for adjusting for possible population stratification in the association analyses [30, 31].

#### Polygenic score (PGS)

In the present study, we measured polygenic predisposition to depressive symptoms using two approaches: (1) the first approach focused on combining the effects of common genetic markers for depressive symptoms only; and (2) the second approach combined the effects of common genetic markers from the traits with an overlapping genetic make-up with depressive symptoms.

Specifically, for the first approach, we calculated PGS for depressive symptoms (PGS-DS) using summary statistics from several GWASs of depressive symptoms conducted in the UK Biobank and GWAS meta-analysis of individuals diagnosed with depressive symptoms and controls with a combined sample of *n* = 1306090 participants [17]. PGS-DS was calculated employing LDpred [31] applied to HapMap3 SNPs as the external linkage disequilibrium (LD) reference sample. This method assumes that SNP effects are drawn from mixtures of distributions with the key parameters defining these architectures estimated through the Bayesian framework [31]. LDpred has been ranked as one of the robust approaches for calculating a polygenic predisposition that maximises the power of PGSs [33], we refer to this PGS as PGS-DS_single_.

For the second approach, to calculate PGS-DS based on multiple traits that have an overlapping genetic make-up with depressive symptoms, genetic correlations between depressive symptoms and 52 traits (related to physical and mental health, behaviours, personality types and educational attainment) were estimated using LD score regressions [17]. This would allow us to identify those traits that have strong genetic correlations with depressive symptoms. These analyses showed that there were substantial genetic correlations between depressive symptoms with subjective well-being (*r*^2^ = −0.776), neuroticism (*r*^2^ = 0.769), loneliness (*r*^2^ = 0.776), and self-rated health (*r*^2^ = −0.614) [17]. Having employed the Multi-Trait Analysis of GWAS (MTAG) approach [23], the GWAS summary statistics for these correlated traits were combined into multi-trait summary statistics for depressive symptoms by conducting a meta-analysis. MTAG was chosen because it can be applied to GWAS summary statistics from an arbitrary number of traits. Because many GWAS summary statistics are likely to have overlapping samples, to account for this potential sample overlap between the GWAS results for different traits, MTAG uses bivariate LD score regression [23]. The combined multi-trait summary statistics were then used to calculate PGS-DS_multi-trait_ using LDpred as described above. To aid the interpretability of the results, PGS-DS_single_ and PGS-ADS_multi-trait_ were standardized to a mean of 0 (standard deviation (SD) = 1).

### Statistical analysis

#### Dealing with missing values

Some variables had missing values (Supplementary Table 3), which, through reduction of the sample size, are likely to lead to the reduced precision of confidence intervals, weakened statistical power and the biased parameter estimates [34, 35]. In ELSA, socio-economic variables are the main drivers of attrition [25], so the assumption that missingness was not dependent on unobserved values (i.e., missing at random (MAR)) was likely to be met). We imputed missing values employing *missForest* [36]. *missForest* is an iterative imputation method based on Random Forests that handles continuous and categorical variables equally well and accommodates non-linear relation structures. *MissForest* has been shown to outperform the well-known imputation methods, such as *k*-nearest neighbours and parametric multivariate imputation by chained equations [36, 37]. This approach handles continuous and categorical variables equally well and accommodates non-linear relation structures [36, 37]. The imputation yielded a minimal error for continuous variables (Normalized Root Mean Squared Error = 0.03%) and categorical variables (proportion of falsely classified = 0.26%) [36, 37]; the distribution of the variables before and after imputation further demonstrated that the imputed values were very closely aligned with the observed values (Supplementary Table 4).

#### Association analyses and interactions

To assess the relationships of PGSs with depressive symptoms at baseline and the rate of change in depressive symptoms during the 14-year follow-up, we employed linear mixed effect models (LMMs) with maximum likelihood estimation [38]. LMMs with maximum likelihood estimation are nonparametric regression models for handling grouped, nested and hierarchical data [39]. LMMs with maximum likelihood estimation models maximise the use of longitudinal data, adjust for the correlation between repeated measures, weight estimates for missing data between waves, and increase statistical power and precision [40]. Having considered linear, quadratic, and cubic LMMs, Akaike Information Criterion and Bayesian Information Criterion [41, 42] showed that the linear model was the most appropriate for our analyses. Interactions between PGSs and socio-economic factors were investigated using multiplicative models, according to which the combined effect of risk factors differs from the product of their individual effects [43]. In terms of correction for multiple testing, it has been emphasized that adjustments for multiple testing are required in confirmatory studies whenever results from multiple tests have to be combined in one final conclusion[44]. Because this study was not a confirmatory study, adjusting our results for multiple testing was not necessary; instead, we presented confidence intervals, which is in line with the new guidelines for statistical reporting [44]. All association analyses were conducted in STATA release 14 (STATACorpLP, USA); *p* ≤ 0.05 were considered statistically significant.

#### Sensitivity analyses

To investigate the impact of missing data imputation, all association analyses were repeated as described above using complete cases only (meaning the data were not imputed). While there was a linear change in depression scores increasing over the 14-year follow-up, the depressive symptoms measured at wave 8 of data collection were substantially higher compared to all other waves of data collection (Supplementary Figure 1). Thus, to investigate the consistency of our findings, we repeated all association analyses excluding the depressive symptoms measured at wave 8 from statistical analysis.

## Results

### Sample characteristics

**Fig. 1:**
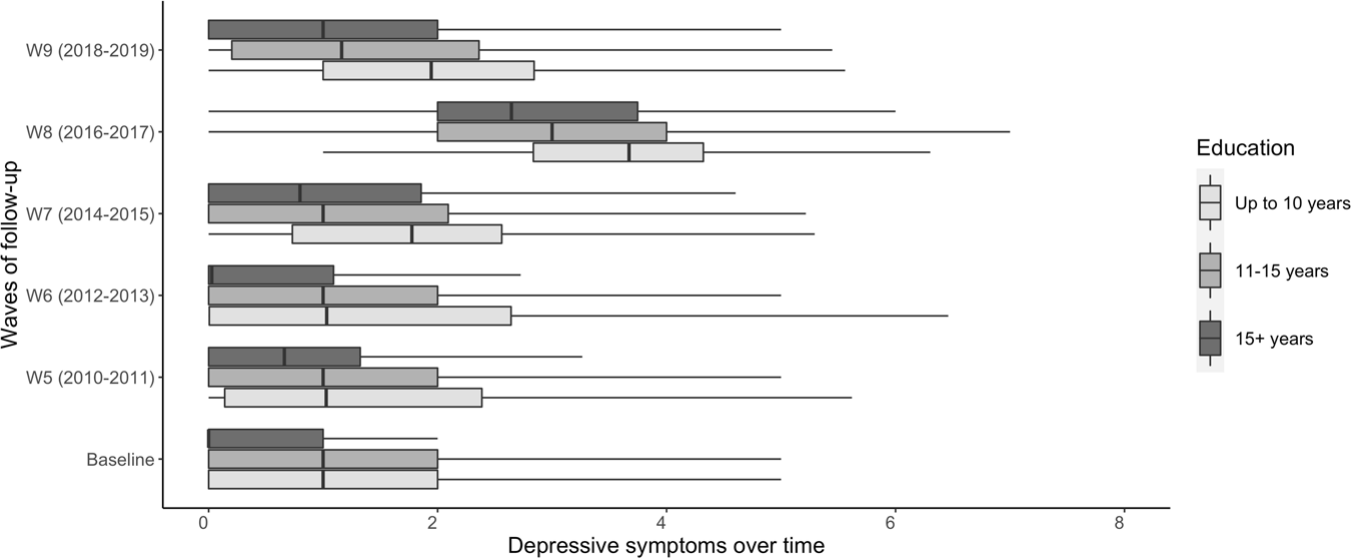
The average distribution of depressive symptoms across all waves of data collection over the 14-year follow-up stratified by years of completed schooling at baseline. W5-W9 symbols stand for the wave number of the follow-up.

**Fig. 2:**
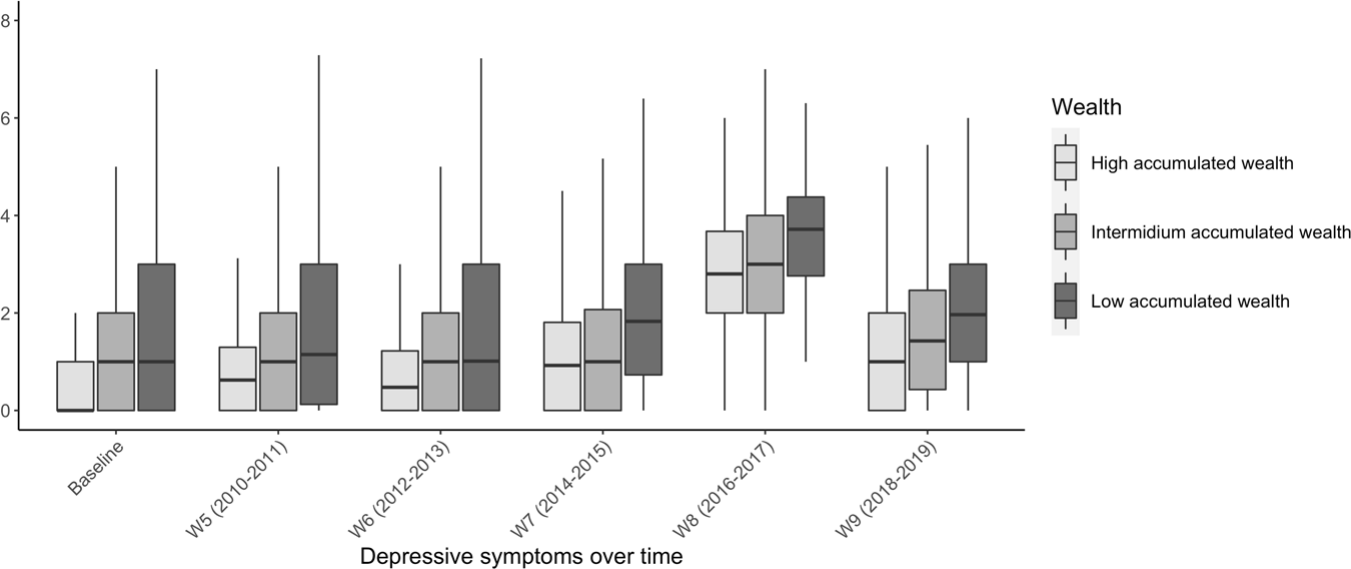
The average distribution of depressive symptoms across all waves of data collection over the 14-year follow-up stratified by wealth at baseline. W5-W9 symbols stand for the wave number of the follow-up.

The sample encompassed 6202 ELSA participants with an average age of 65.2 years old (standard deviation (SD) = 9.7, median = 64, IQR = 57–72). Of these, 47.8% (*n* = 2964) were men and 35.5% (*n* = 2181) had a low wealth (Table 1). The mean level of educational attainment in the entire sample was 13.9 years (SD = 3.7). The average baseline depressive symptoms were 1.29 (SD = 1.73). Older adults with lower education attainment showed to have higher depressive symptoms during the 14-year follow-up period (Fig. 1). Similarly, older adults with a low wealth had higher average depressive scores during the follow-up compared to adults with higher wealth (Fig. 2). Further, compared to men, women had higher average depressive symptoms scores for each wave of data collection (Supplementary Table 5).

**Table 1:** Sample characteristics at baseline.

Baseline sample characteristics	Total sample (*n* = 6202)	
	*n*/Mean	Frequency (%)/SD
Age at baseline (years)	65.2	9.7
Median (IQR)	64	57 to 72
*Gender*		
Female	3238	52.2
Male	2964	47.8
*Accumulated wealth*		
Low	2181	35.5
Intermediate	1701	27.6
High	2272	36.9
Educational attainment (years)	13.9	3.7
Depressive symptoms	1.3	1.7

*SD* standard deviation, *IQR* the interquartile range.

### PGS-DS_single_, educational attainment, and wealth

One standard deviation (1-SD) increase in PGS-DS_single_ was associated with higher baseline depressive symptoms by an average of 0.35 points (95%CI = 0.27 to 0.44, *p* < 0.001) (Table 2). Each additional year of completed schooling was associated with a lower baseline score in depression symptoms (*β* = −0.06, 95%CI = −0.07 to −0.05, *p* < 0.001). Intermediate and low wealth were shown to associate with a higher baseline score in depressive symptoms by an average of 0.31 and 0.77 points, respectively (Table 3). There was an interaction between PGS-DS_single_ and educational attainment (*β* = −0.01, 95%CI = −0.02 to −0.01, *p* < 0.001) where each additional year of completed schooling was associated with a reduction in depressive symptoms by an average of 0.01 point among those adults who had a high polygenic predisposition to depressive symptoms (Table 2). A significant multiplicative interaction effect between PGS-DS_single_ and a low level of wealth in association with the baseline depressive symptoms (*β* = 0.08, 95%CI = 0.03 to 0.13, *p* = 0.002) highlighted that 1-SD increase in PGS-DS_single_ was associated with an increase in the number of depressive symptoms by 0.08 points in adults with a low wealth (Table 3).

**Table 2:** Longitudinal mixed models exploring the main effect of polygenic score for DS and educational attainment, and interaction between these two variables, in relation to depressive symptoms trajectories during the 14-year follow-up period.

	Polygenic score for DS_single_			Polygenic score for DS_multi_		
	*β* (SE)	95% CI	*P*	*β* (SE)	95% CI	*P*
*Baseline*						
PGS	0.35 (0.04)	0.27 to 0.44	<0.001	0.31 (0.04)	0.23 to 0.40	<0.001
Years of completed schooling	−0.06 (0.004)	−0.07 to −0.05	<0.001	−0.06 (0.004)	−0.06 to −0.05	<0.001
PGS × Years of completed schooling	−0.01 (0.003)	−0.02 to −0.01	<0.001	−0.01 (0.003)	−0.01 to −0.003	0.004
*Rate of change*						
PGS	−0.02 (0.01)	−0.04 to 0.01	0.201	−0.01 (0.01)	−0.04 to 0.01	0.242
Years of completed schooling	0.001 (0.001)	−0.001 to 0.004	0.312	0.00 (0.001)	−0.002 to 0.004	0.350
PGS × Years of completed schooling	0.00 (0.00)	−0.00 to 0.00	0.494	0.00 (0.00)	−0.00 to 0.00	0.698
*Variance*^a^	0.06 (0.002)	0.06 to 0.07		0.06 (0.002)	0.06 to 0.07	

× represents an interaction between the two factors; interactions are presented based on multiplicative interaction model.

*PGS* polygenic score, *DS* depressive symptoms, *CI* confidence intervals, *SE* standard errors.

^a^The estimated variance captured random or stochastic variability in the data that comes from participants, here showing that there were significant individual differences in measure of depressive symptoms over time.

The models are adjusted for sex, age and genetic ancestry as captured by 10 principal components; to capture the non-linear effects of ageing, we further included age^2^ as a covariate.

**Table 3:** Longitudinal mixed models exploring the main effect of polygenic score for DS and accumulated wealth, and interaction between these two variables, in relation to depressive symptoms trajectories during the 14-year follow-up period.

	Polygenic score for DS_single_			Polygenic score for DS_multi_		
	*β* (SE)	95% CI	*P*	*β* (SE)	95% CI	*P*
*Baseline*						
PGS	0.12 (0.02)	0.08 to 0.17	<0.001	0.13 (0.02)	0.09 to 0.17	<0.001
Accumulated wealth—moderate	0.31 (0.03)	0.25 to 0.37	<0.001	0.30 (0.03)	0.24 to 0.37	<0.001
Accumulated wealth—low	0.77 (0.03)	0.71 to 0.83	<0.001	0.76 (0.03)	0.70 to 0.82	<0.001
PGS × Accumulated wealth—moderate	0.03 (0.03)	−0.02 to 0.09	0.204	0.05 (0.03)	−0.004 to 0.10	0.070
PGS × Accumulated wealth—low	0.08 (0.03)	0.03 to 0.13	0.002	0.09 (0.03)	0.03 to 0.14	0.001
*Rate of change*						
PGS	−0.004 (0.01)	−0.02 to 0.12	0.659	−0.01 (0.01)	−0.02 to 0.01	0.523
Accumulated wealth—moderate	−0.01 (0.01)	−0.03 to 0.02	0.628	−0.01 (0.01)	−0.03 to 0.02	0.648
Accumulated wealth—low	−0.02 (0.01)	−0.05 to 0.001	0.063	−0.02 (0.01)	−0.05 to 0.002	0.076
PGS × Accumulated wealth—moderate	−0.001 (0.002)	−0.01 to 0.00	0.820	−0.001 (0.002)	−0.01 to 0.004	0.778
PGS × Accumulated wealth—low	−0.002 (0.002)	−0.01 to 0.00	0.366	−0.002 (0.002)	−0.01 to 0.002	0.352
*Variance*^a^	0.06 (0.002)	0.06 to 0.07		0.06 (0.002)	0.06 to 0.07	

× represents an interaction between the two factors; interactions are presented based on multiplicative interaction model.

*PGS* polygenic score, *DS* depressive symptoms, *CI* confidence intervals, *SE* standard errors.

^a^The estimated variance captured random or stochastic variability in the data that comes from participants, here showing that there were significant individual differences in measure of depressive symptoms over time.

The models are adjusted for sex, age and genetic ancestry as captured by 10 principal components; to capture the non-linear effects of ageing, we further included age^2^ as a covariate.

### PGS-DS_multi-trait_, educational attainment, and wealth

1-SD increase in PGS-DS_multi-trait_ was associated with a higher number of baseline depressive symptoms (*β* = 0.31, 95%CI = 0.23 to 0.40, *p* < 0.001) (Table 2). Independently from PGS-DS_multi-trait_, each additional year of completed schooling was associated with a lower baseline score of depression symptoms by an average of 0.06 points (95%CI = −0.07 to −0.05, *p* < 0.001) (Table 2); whereas intermediate and low levels of wealth were associated with higher baseline depressive symptoms by an average 0.30 and 0.76 points, respectively (Table 3). There was a weak but significant interaction effect between PGS-DS_multi-trait_ with years of completing schooling (*β* = −0.01, 95%CI = −0.01 to −0.003, *p* = 0.004), which indicated that each additional year of completed schooling was associated with a reduction in depressive symptoms by an average 0.01 point among older adults with a higher polygenic predisposition to depressive symptoms as measured with a multi-trait PGS (Table 2). Similarly, there was a multiplicative interaction effect between PGS-DS_multi-trait_ and lower wealth (*β* = 0.09, 95%CI = 0.03 to 0.14, *p* = 0.001) in association with the baseline depressive symptoms highlighting that 1-SD increase in PGS-DS_multi-trait_ was associated with an increase in the number of depressive symptoms by 0.09 points in adults with low wealth (Table 3). However, there were no significant associations between PGS-DS_multi-trait_, socio-economic factors, and rate of change in the depressive symptoms during the 14-year follow-up period.

### Sensitivity analyses

When using the complete case only (i.e., unimputed data) (Supplementary Tables 6–7), and once we excluded the depressive symptoms measured at wave 8 (Supplementary Tables 8–9), our results remained largely unchanged.

## Discussion

To our knowledge, this is the first study to investigate the relationships of a polygenic predisposition to depressive symptoms, as measured with a single trait and multi-trait approaches, with individual differences in depressive symptoms at baseline and a rate of change in depressive symptoms in the following 14 years adults aged 50 years old and older. Cumulatively, our results contribute to a better understanding of the role a higher polygenic predisposition to depressive symptoms, independently or in interaction with socio-economic status, which was measured by educational attainment and wealth, plays in increasing risk for depressive symptoms onset and their longitudinal trajectory in older adults, for which knowledge is currently lacking.

Our results showed that the risk of having higher depressive symptoms is amplified by a higher loading of common genetic markers associated with depressive symptoms, with a greater genetic liability indicating a greater risk in older adults. Building on previous findings showing that PGS for depression derived from adults generalizes to depression outcomes including depressive symptoms severity in youths [18] and middle-aged adults [14], these results may suggest that individual differences in depressive symptoms are influenced by an individual load of common genetic markers associated with depressive symptoms throughout a lifespan. Even though a multi-trait polygenic score maximises the predictive power for depression [23], comparing the effect sizes (the beta coefficients and standard errors from the models) our results suggest that a polygenic score for depressive symptoms that encompass genetic information from the related traits, such as subjective well-being, neuroticism, loneliness, and self-rated health in addition to depressive symptoms, was not a stronger predictor of depressive symptoms at baseline and a rate of change in depressive symptoms in the following 14 years when compared to PGS_single-trait_. This result is unexpected considering there is evidence suggesting that each of the traits included in the multi-trait polygenic score was shown to associate with depressive symptoms [45]. This may suggest that the multi-trait associations are due to the inclusion of depressive symptoms traits within the combined score and that the additional factors make no difference. Nonetheless, this mapping of aetiological sources of cross-disorder overlap can guide future research aiming to identify specific mechanisms contributing to the risk of depressive symptoms onset in older adults from the general population.

In agreement with a consensus that higher educational attainment is protective against the onset of depressive symptoms [6, 46], our results showed that each additional year of completed schooling was associated with a lower score in depressive symptoms at baseline in older adults. It has been hypothesised that higher educational attainment may protect from the depressive symptom risk via more effective coping strategies or healthier lifestyles [6, 47, 48]. Similarly, a lower wealth, which reflects limited socio-economic resources available later in life, low digital literacy, and limited access to participation in cultural activities or reduced social networks [49, 50], was also highlighted to be an important factor influencing individual levels of depressive symptoms independently from polygenic predisposition to depressive symptoms. Because educational attainment and wealth are indicators of socio-economic position achieved during different stages of a person’s life [10], they are likely to have different impacts on person’s life, health, and well-being. This is further reflected in our results where the direct effect of the PGS on depression onset decreased by 50% in analyses that included wealth and not educational attainment. This result reiterates that different mechanisms influence depression onset depending on socio-economic status.

We further observed an interaction effect between polygenic predisposition to depressive symptoms and low socio-economic status in association with the onset of depressive symptoms in older adults. These results are supported by a twin study highlighting shared genetic risk for low education and major depression [48]. Therefore, it should be a priority for policymakers to make the best efforts to reduce socio-economic inequalities [51]. Additionally, support from mental health professional by providing psychoeducation on coping strategies and stress reduction [52] associated with having lower educational attainment and wealth [53], especially among older adults who have a higher polygenic predisposition to depressive symptoms, may prove beneficial in reducing the risk for developing depressive symptoms in older adults.

Although an increase in depressive symptoms was observed over the 14-year-long follow-up time, common genetic variants associated with depressive symptoms additively were not associated with a greater increase in depressive symptoms during this period in older adults from the general population. We further did not observe a significant interaction between indicator of lower socio-economic status and polygenic predisposition in influencing the rate of change in depressive symptoms during 14 years of follow-up. These non-significant findings are in line with findings from studies conducted in US and Japan [54, 55]. For example, in the Chicago Health and Aging Project encompassing 4275 community-dwelling individuals aged 65 and older, there did not appear to be consistent changes in depressive symptoms over the 9-year follow-up [55]. Nonetheless, the non-significant results may also reflect attrition effects, which are unavoidable in longitudinal cohorts. Similarly, because the results presented in the study are based on a longitudinal study with prospectively collected data, collider bias may have contributed to the non-significant findings [53], which might have arisen from selection bias or attrition. However, the proportion of missingness in the present study was comparable to many longitudinal cohorts [56–58]; we further imputed missing values using robust approaches [36, 37]. Therefore, it is unlikely that attrition or selection bias influenced our results. It is nevertheless possible that only a subset of the genetic factors for depressive symptoms may have an impact on individual differences in the rate of change in depressive symptoms, which, due to the nature of the PGS approach, might not have been captured in the present study. Therefore, further analyses, such as pathway-specific polygenic score analyses, genomic structural equation modelling and gene-set enrichment analyses, may be needed before we can draw more conclusions.

### Methodological considerations

This study consisted of a large sample size, which was a national cohort of older adults from England followed up for 14 years. It included a relatively equal proportion of men and women with diverse backgrounds and different socio-economical positions. The duration of the study brings a great advantage of repeated measures for depressive symptoms over 14 years of follow-up. Nonetheless, even though PGS is a good marker for genetic risk and a tool for studying gene-environment interactions, it may have poor generalisability across populations because results are mostly based on European participants. Further studies are needed to assess genetic risk, develop PGS models and assess gene-environment interactions for populations with non-European ancestry. To minimise chances of collider bias affecting our findings [59], covariates in the present study were those that were set at birth; however, we did not adjust the confounding effect of other factors on individual differences in depressive symptoms at baseline and during the follow-up. Although it is feasible that PGS for depressive symptoms utilised in this study might have incorporated genetic effects on educational attainment and wealth, evidence suggests that the genetic correlation between the polygenic score for depression and educational attainment is small (*r*^2^ = −0.25) [23]. To our knowledge, a polygenic score for wealth has not been ascertained yet; therefore, it is not possible to know if, and to what degree, depression and wealth may be genetically correlated. Similarly, given the high comorbidity of depression and anxiety [60], it is possible that PGS for depression might have incorporated the common genetic effects of anxiety disorders. The issues of missing data are unavoidable in research, especially with longitudinal study design. The proportion of missingness in the present study was as expected for any longitudinal population-based cohorts and comparable to many longitudinal cohorts [56, 57]. Finally, as the group comparisons highlighted, the analytic sample included in the analyses was not fully representative of the entire ELSA cohort and thus some causation is needed when generalising the findings.

### Conclusion

Polygenic predisposition to depressive symptoms was associated with higher levels of depressive symptoms in older adults; though, this risk was not better captured by incorporating genetic information from correlated traits. Lower socio-economic status is also an important factor influencing individual levels of depressive symptoms, independently from polygenic predisposition to depressive symptoms in older adults. Therefore, providing psychoeducation about how to reduce the stress associated with having lower educational attainment and wealth, especially among older adults who have a higher polygenic predisposition to depressive symptoms, may prove beneficial in reducing the risk of developing depressive symptoms in older adults. However, a polygenic predisposition to depressive symptoms was not associated with the rate of change in depressive symptoms during a 14-year follow-up highlighting other factors that may be important contributors to the change in depressive symptoms over time.

## Supplementary information

Supplementary materials

## Data Availability

The English Longitudinal Study of Ageing (ELSA) was developed by a team of researchers based at University College London, the Institute for Fiscal Studies and the National Centre for Social Research. The datasets generated and/or analysed during the current study are available in UK Data Services and can be accessed at: https://discover.ukdataservice.ac.uk. No administrative permissions were required to access these data.
