# Supplementary material for: Genetic propensity, socioeconomic status, and trajectories of depression over a course of 14 years in older adults
Source: Transl Psychiatry. 2023 Feb 23;13:68. doi: 10.1038/s41398-023-02367-9 (PMC9950051; doi:10.1038/s41398-023-02367-9)
Supplement: Supplementary file 1 — Supplementary materials [file 41398_2023_2367_MOESM1_ESM.docx]

**Supplementary Figure 1. Depicts a distribution of the depressive symptoms for each wave of data collection**

**
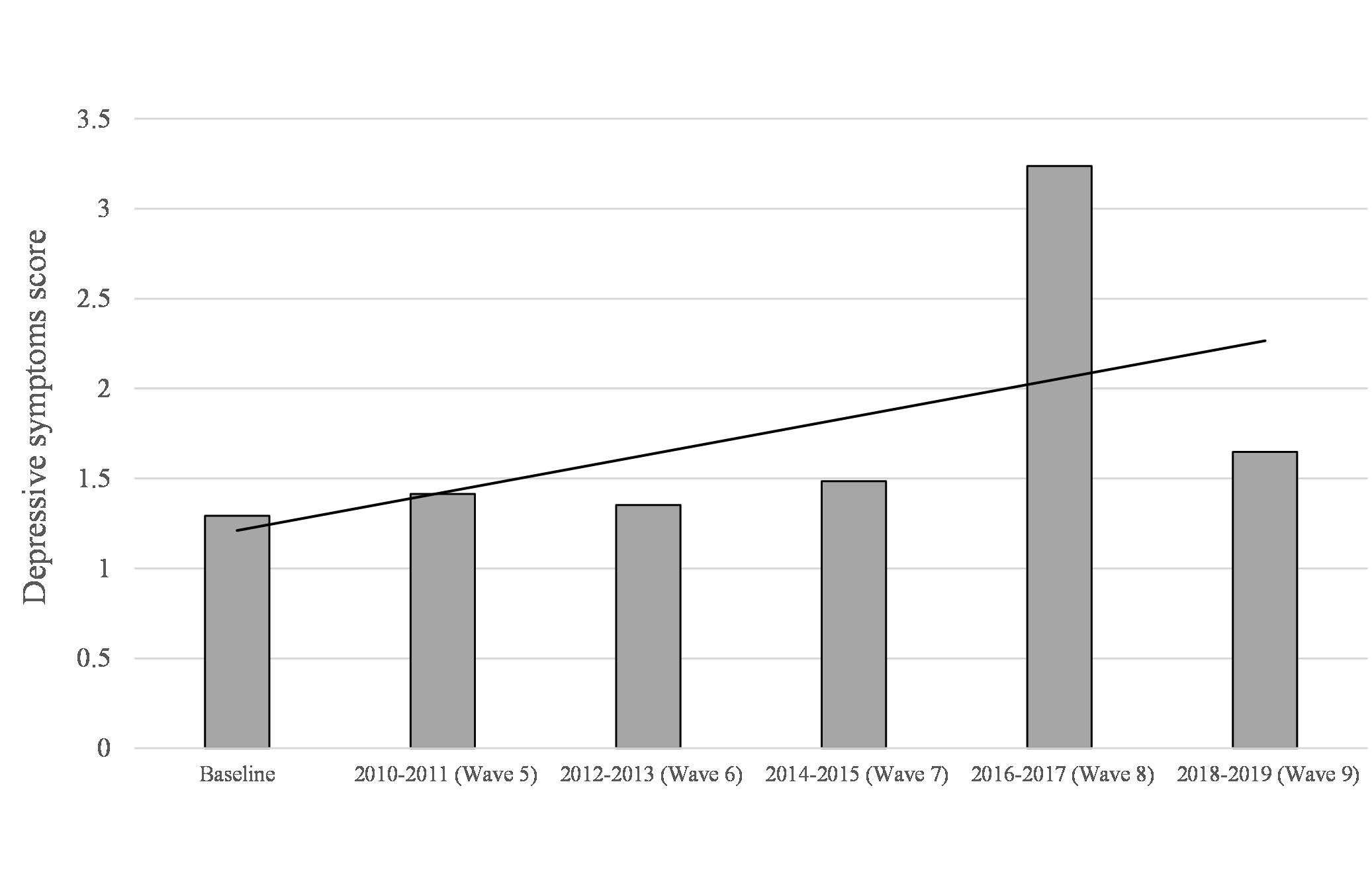
**

**Supplementary Table 1. Comparisons between the ELSA participants who were included in the analyses and those who were excluded**

|  |  |  | **Excluded (N=3340)** | **Included (N=6202)** |  |
| --- | --- | --- | --- | --- | --- |
|  |  |  | Mean (SD) / N(%) | Mean (SD) / N(%) | Statistics |
| *Socio-demographic characteristics* | |  |  |  |  |
|  | Age (years) |  | 62.0 (9.1) | 65.2 (9.7) | *t*=-16.1, df= 7245, p<0.001 |
|  | Sex (male) |  | 1383 (41.4) | 2964 (47.8) | *x*^2^=35.4, df=1, p<0.001 |
|  | No qualification |  | 965 (28.9) | 2153 (34.7) | *x^2^*=46.7, df=6, p<0.001 |
|  | Accumulated wealth |  |  |  | *x*^2^=60.5, df=2, p<0.001 |
|  | High |  | 1097 (33.4) | 2272 (36.9) |  |
|  | Intermediate |  | 1161 (35.4) | 1701 (27.6) |  |
|  | Low |  | 1026 (31.2) | 2181 (35.5) |  |
|  |  |  |  |  |  |
| *Comorbid health issues* | |  |  |  |  |
|  | Limiting health conditions |  | 912 (27.3) | 1335 (21.5) | *x*^2^=40.2, df=1, p<0.001 |
|  | Depressive symptoms |  | 644 (19.3) | 764 (12.3) | *x^2^*=83.7, df=1, p<0.001 |
|  |  |  |  |  |  |
| *Behavioural outcomes* | |  |  |  |  |
|  | Currently a smoker |  | 595 (17.8) | 955 (15.4) | *x*^2^=9.1, df=1, p=0.003 |

**Supplementary Table 2. Distribution of the depressive symptoms for each wave of data collection for all ELSA participants included in this study**

| **Depressive symptoms** |  | **Baseline** | **Wave 5:**  **2010-2011** | **Wave 6: 2012-2013** | **Wave 7:**  **2014-2015** | **Wave 8: 2016-2017** | **Wave 9:**  **2018-2019** |
| --- | --- | --- | --- | --- | --- | --- | --- |
|  |  |  |  |  |  |  |  |
| Mean (SD) |  | 1.29 (1.73) | 1.41 (1.70) | 1.35 (1.65) | 1.48 (1.58) | 3.23 (1.16) | 1.64 (1.44) |
| Median (IQR) |  | 1 (0 to 2) | 1 (0 to 2) | 0 (0 to 2) | 1 (0 to 2) | 2 (2 to 3) | 1 (0 to 2) |
| N |  | 6202 | 4600 | 4231 | 3708 | 3242 | 2887 |

SD, standard deviation; ELSA, English Longitudinal Study of Ageing

**Supplementary Table 3. Distribution of missing and observed variables at baseline and follow-up in ELSA**

| **Variables included in the analyses** | | **N observed** | **% observed** | **N missing** | **% missing** |
| --- | --- | --- | --- | --- | --- |
|  | Age | 6161 | 99.3 | 41 | 0.7 |
|  | Educational attainment | 6161 | 99.3 | 41 | 0.7 |
|  | Sex | 6161 | 99.3 | 41 | 0.7 |
|  | Accumulated wealth | 6161 | 99.3 | 41 | 0.7 |
|  | Depressive symptoms at baseline | 6202 | 100 | 0 | 0.0 |
|  | Depressive symptoms at wave 5 | 4600 | 74.2 | 1602 | 25.8 |
|  | Depressive symptoms at wave 6 | 4231 | 68.2 | 1971 | 31.8 |
|  | Depressive symptoms at wave 7 | 3708 | 59.8 | 2494 | 40.2 |
|  | Depressive symptoms at wave 8 | 3242 | 52.3 | 2960 | 47.7 |
|  | Depressive symptoms at wave 9 | 2887 | 46.5 | 3315 | 53.5 |

**Supplementary Table 4. Distribution of variables at baseline before and after imputation**

| **Variables included in the analyses** | | **Before imputation** |  | **After imputation** |
| --- | --- | --- | --- | --- |
|  |  | N (%) /Mean (SD) |  | N (%) /Mean (SD) |
|  | Age | 65.4 (9.8) |  | 65.4 (9.8) |
|  | Educational attainment | 13.9 (3.7) |  | 13.9 (3.7) |
|  | Male | 2957 (48.0) |  | 2961 (47.7) |
|  | Female | 3204 (52.0) |  | 3241 (52.3) |
|  | Levels of wealth- Low | 2224 (36.1) |  | 2238 (36.1) |
|  | Levels of wealth- Intermediate | 1898 (30.8) |  | 1910 (30.8) |
|  | Levels of wealth- High | 2039 (33.1) |  | 2054 (33.1) |
|  | Depressive symptoms at baseline | 1.3 (1.7) |  | 1.2 (1.6) |
|  | Depressive symptoms at wave 5 | 1.3 (1.7) |  | 1.4 (1.7) |
|  | Depressive symptoms at wave 6 | 1.1 (1.7) |  | 1.3 (1.6) |
|  | Depressive symptoms at wave 7 | 1.1 (1.6) |  | 1.5 (1.6) |
|  | Depressive symptoms at wave 8 | 2.9 (1.2) |  | 3.2 (1.3) |
|  | Depressive symptoms at wave 9 | 1.2 (1.6) |  | 1.6 (1.4) |

**Supplementary Table 5. Comparisons of the average depressive symptoms for each wave of data collection between men and women in the ELSA sample included in the analyses**

| **Depressive symptoms** |  | **Women** |  | **Men** |  | **Test statistics** |
| --- | --- | --- | --- | --- | --- | --- |
|  |  | Mean (SD) |  | Mean (SD) |  | **t, SD, p** |
| **Baseline** |  | 1.56 (1.89) |  | 0.99 (1.49) |  | 13.07, 6200, <0.001 |
| **Wave 5: 2010-2011** |  | 1.65 (1.82) |  | 1.15 (1.50) |  | 11.86, 6200, <0.001 |
| **Wave 6: 2012-2013** |  | 1.60 (1.78) |  | 1.08 (1.43) |  | 12.53, 6200, <0.001 |
| **Wave 7: 2014-2015** |  | 1.70 (1.67) |  | 1.25 (1.44) |  | 11.42, 6200, <0.001 |
| **Wave 8: 2016-2017** |  | 3.41(1.21) |  | 3.05 (1.07) |  | 12.10, 6200, <0.001 |
| **Wave 9: 2018-2019** |  | 1.89 (1.56) |  | 1.38 (1.25) |  | 14.13, 6200, <0.001 |

SD, standard deviation; ELSA, English Longitudinal Study of Ageing

**Supplementary Table 6. Longitudinal mixed models investigating the relationship of Polygenic score for D**S**_single_ and Polygenic score for D**S**_multi_ with educational attainment, and interaction between these two variables, in relation to depressive symptoms trajectories during the 14-year follow-up period using complete case data (i.e., unimputed data).**

|  |  |  | Polygenic score for DS _single_ | | |  | Polygenic score for DS _multi_ | | |
| --- | --- | --- | --- | --- | --- | --- | --- | --- | --- |
|  |  |  | **β (SE)** | **95% CI** | **P** |  | **β (SE)** | **95% CI** | **P** |
|  | |  |  |  |  |  |  |  |  |
| *Baseline* | |  |  |  |  |  |  |  |  |
|  | PGS |  | 0.34 (0.05) | 0.24- 0.44 | <0.001 |  | 0.31 (0.05) | 0.21- 0.41 | <0.001 |
|  | Years of completed schooling |  | -0.06 (0.004) | -0.07 to -0.05 | <0.001 |  | -0.05 (0.004) | -0.06 to -0.05 | <0.001 |
|  | PGS × Years of completed schooling |  | -0.01 (0.003) | -0.02 to –0.01 | 0.001 |  | -0.01 (0.003) | -0.01 to –0.002 | 0.013 |
|  |  |  |  |  |  |  |  |  |  |
| *Rate of change* | |  |  |  |  |  |  |  |  |
|  | PGS |  | -0.01 (0.02) | -0.04 to 0.02 | 0.642 |  | -0.01 (0.02) | -0.04 to 0.02 | 0.636 |
|  | Years of completed schooling |  | 0.003 (0.002) | -0.001 to 0.01 | 0.157 |  | 0.003 (0.002) | -0.001 to 0.01 | 0.174 |
|  | PGS × Years of completed schooling |  | 0.00 (0.0002) | -0.00 to 0.001 | 0.738 |  | 0.00 (0.0002) | -0.00 to 0.00 | 0.934 |
|  |  |  |  |  |  |  |  |  |  |
|  |  |  |  |  |  |  |  |  |  |
| *Variance* | |  | 0.07 (0.003) | 0.06-0.07 |  |  | 0.07 (0.003) | 0.06-0.07 |  |

PGS, polygenic score; DS, depressive symptoms; CI, confidence intervals

× represents an interaction between the two factors; interactions are presented based on multiplicative interaction model

^a^ the estimated variance captured random or stochastic variability in the data that comes from participants, here showing that there were significant individual differences in measure of depressive symptoms over time.

The models are adjusted for sex, age and genetic ancestry as captured by 10 principal components; to capture the non-linear effects of ageing, we further included age2 as a covariate.

**Supplementary Table 7.** **Longitudinal mixed models investigating the relationship of Polygenic score for D**S **_single_ and Polygenic score for D**S **_multi_ with accumulated wealth, and interaction between these two variables, in relation to depressive symptoms trajectories during the 14-year follow-up period using complete case data (i.e., unimputed data).**

|  |  |  | **Polygenic score for MDD _single_** | | |  | **Polygenic score for MDD _multi_** | | |
| --- | --- | --- | --- | --- | --- | --- | --- | --- | --- |
|  |  |  | **β (SE)** | **95% CI** | **P** |  | **β (SE)** | **95% CI** | **P** |
| *Baseline* | |  |  |  |  |  |  |  |  |
|  | PGS |  | 0.12 (0.03) | 0.08-0.17 | <0.001 |  | 0.13 (0.03) | 0.08-0.18 | <0.001 |
|  | Accumulated wealth - moderate |  | 0.30 (0.04) | 0.22-0.37 | <0.001 |  | 0.29 (0.04) | 0.22-0.37 | <0.001 |
|  | Accumulated wealth - low |  | 0.70 (0.04) | 0.63-0.78 | <0.001 |  | 0.69 (0.04) | 0.62-0.77 | <0.001 |
|  |  |  |  |  |  |  |  |  |  |
|  | PGS × Accumulated wealth - moderate |  | 0.05 (0.03) | -0.02 to 0.11 | 0.140 |  | 0.06 (0.03) | -0.01 to 0.12 | 0.078 |
|  | PGS × Accumulated wealth - low |  | 0.06 (0.03) | -0.01 to 0.12 | 0.080 |  | 0.07 (0.03) | 0.01-0.14 | 0.029 |
|  |  |  |  |  |  |  |  |  |  |
| *Rate of change* | |  |  |  |  |  |  |  |  |
|  | PGS |  | -0.003 (0.01) | -0.02 to 0.02 | 0.804 |  | -0.004 (0.01) | -0.02 to 0.02 | 0.690 |
|  | Accumulated wealth - moderate |  | -0.01 (0.02) | -0.05 to 0.02 | 0.447 |  | -0.01 (0.02) | -0.05 to 0.02 | 0.460 |
|  | Accumulated wealth - low |  | -0.04 (0.02) | -0.07 to 0.001 | 0.044 |  | -0.03 (0.02) | -0.07 to 0.001 | 0.053 |
|  |  |  |  |  |  |  |  |  |  |
|  | PGS × Accumulated wealth - moderate |  | 0.000 (0.003) | -0.01 to 0.01 | 0.953 |  | -0.0004 (0.003) | -0.01 to 0.01 | 0.891 |
|  | PGS × Accumulated wealth - low |  | -0.001 (0.003) | -0.01 to 0.01 | 0.821 |  | -0.001 (0.003) | -0.01 to 0.01 | 0.753 |
|  |  |  |  |  |  |  |  |  |  |
| *Variance ^a^* | |  | 0.06 (0.003) | 0.06-0.07 |  |  | 0.06 (0.003) | 0.06-0.07 |  |

PGS, polygenic score; DS, depressive symptoms; CI, confidence intervals

× represents an interaction between the two factors; interactions are presented based on multiplicative interaction model

^a^ the estimated variance captured random or stochastic variability in the data that comes from participants, here showing that there were significant individual differences in measure of depressive symptoms over time.

The models are adjusted for sex, age and genetic ancestry as captured by 10 principal components; to capture the non-linear effects of ageing, we further included age^2^ as a covariate.

**Supplementary Table 8.**  **Longitudinal mixed models investigating the relationship of Polygenic score for D**S **_single_ and Polygenic score for D**S **_multi_ with years of completed schooling, and interaction between these two variables, in relation to depressive symptoms trajectories during the 14-year follow-up period using the data where the depressive symptoms measured at wave 8 of data collection were removed from the analyses.**

|  |  |  | **Polygenic score for MDD _single_** | | |  | **Polygenic score for MDD _multi_** | | |
| --- | --- | --- | --- | --- | --- | --- | --- | --- | --- |
|  |  |  | **β (SE)** | **95% CI** | **P** |  | **β (SE)** | **95% CI** | **P** |
| *Baseline* | |  |  |  |  |  |  |  |  |
|  | PGS |  | 0.35 (0.04) | 0.26-0.43 | <0.001 |  | 0.30 (0.04) | 0.22-0.39 | <0.001 |
|  | Years of completed schooling |  | -0.06 (0.003) | -0.07 to –0.05 | <0.001 |  | -0.06 (0.003) | -0.06 to –0.05 | <0.001 |
|  | PGS × Years of completed schooling |  | -0.01 (0.003) | -0.02 to –0.01 | <0.001 |  | -0.01 (0.003) | -0.01 to 0.002 | 0.004 |
|  |  |  |  |  |  |  |  |  |  |
| *Rate of change* | |  |  |  |  |  |  |  |  |
|  | PGS |  | -0.002 (0.01) | -0.03 to 0.02 | 0.868 |  | -0.001 (0.01) | -0.02 to 0.02 | 0.938 |
|  | Years of completed schooling |  | 0.001 (0.002) | -0.002 to 0.005 | 0.377 |  | 0.001 (0.002) | -0.002 to 0.005 | 0.400 |
|  | PGS × Years of completed schooling |  | -0.00 (0.0002) | -0.00 to 0.00 | 0.823 |  | -0.00 (0.0002) | -0.00 to 0.00 | 0.562 |
|  |  |  |  |  |  |  |  |  |  |
| *Variance ^a^* | |  | 0.10 (0.003) | 0.10-0.11 |  |  | 0.10 (0.003) | 0.09-0.11 |  |

PGS, polygenic score; DS, depressive symptoms; CI, confidence intervals

× represents an interaction between the two factors; interactions are presented based on multiplicative interaction model

^a^ the estimated variance captured random or stochastic variability in the data that comes from participants, here showing that there were significant individual differences in measure of depressive symptoms over time.

The models are adjusted for sex, age and genetic ancestry as captured by 10 principal components; to capture the non-linear effects of ageing, we further included age2 as a covariate.

**Supplementary Table 9.** **Longitudinal mixed models investigating the relationship of Polygenic score for D**S **_single_ and Polygenic score for D**S**_multi_ with accumulated wealth, and interaction between these two variables, in relation to depressive symptoms trajectories during the 14-year follow-up period using the data where the depressive symptoms measured at wave 8 of data collection were removed from the analyses.**

|  |  |  | **Polygenic score for MDD _single_** | | |  | **Polygenic score for MDD _multi_** | | |
| --- | --- | --- | --- | --- | --- | --- | --- | --- | --- |
|  |  |  | **β (SE)** | **95% CI** | **P** |  | **β (SE)** | **95% CI** | **P** |
| *Baseline* | |  |  |  |  |  |  |  |  |
|  | PGS |  | 0.12 (0.02) | 0.08 to 0.16 | <0.001 |  | 0.13 (0.02) | 0.09 to 0.17 | <0.001 |
|  | Accumulated wealth - moderate |  | 0.31 (0.03) | 0.25 to 0.37 | <0.001 |  | 0.30 (0.03) | 0.24 to .36 | <0.001 |
|  | Accumulated wealth - low |  | 0.76 (0.03) | 0.71 to 0.82 | <0.001 |  | 0.75 (0.03) | 0.69 to 0.81 | <0.001 |
|  |  |  |  |  |  |  |  |  |  |
|  | PGS × Accumulated wealth - moderate |  | 0.04 (0.03) | -0.02 to 0.09 | 0.174 |  | 0.05 (0.03) | -0.001 to 0.10 | 0.054 |
|  | PGS × Accumulated wealth - low |  | 0.08 (0.03) | 0.03 to 0.13 | 0.002 |  | 0.09 (0.03) | 0.04 to 0.14 | 0.001 |
|  |  |  |  |  |  |  |  |  |  |
| *Rate of change* | |  |  |  |  |  |  |  |  |
|  | PGS |  | -0.00 (0.01) | -0.02 to 0.02 | 0.959 |  | -0.002 (0.01) | -0.02 to 0.01 | 0.792 |
|  | Accumulated wealth - moderate |  | -0.003 (0.01) | -0.03 to 0.02 | 0.829 |  | -0.003 (0.01) | -0.03 to 0.02 | 0.848 |
|  | Accumulated wealth - low |  | -0.02 (0.01) | -0.04 to 0.01 | 0.286 |  | -0.01 (0.01) | -0.04 to 0.01 | 0.308 |
|  |  |  |  |  |  |  |  |  |  |
|  | PGS × Accumulated wealth - moderate |  | -0.001 (0.002) | -0.01 to 0.004 | 0.773 |  | -0.0004 (0.002) | -0.01 to 0.004 | 0.856 |
|  | PGS × Accumulated wealth - low |  | -0.002 (0.002) | -0.01 to 0.003 | 0.465 |  | -0.001 (0.002) | -0.01 to 0.003 | 0.558 |
|  |  |  |  |  |  |  |  |  |  |
| *Variance ^a^* | |  | 0.10 (0.003) | 0.09-0.10 |  |  | 0.10 (0.003) | 0.09-0.10 |  |

PGS, polygenic score; DS, depressive symptoms; CI, confidence intervals

× represents an interaction between the two factors; interactions are presented based on multiplicative interaction model

^a^ the estimated variance captured random or stochastic variability in the data that comes from participants, here showing that there were significant individual differences in measure of depressive symptoms over time.

The models are adjusted for sex, age and genetic ancestry as captured by 10 principal components; to capture the non-linear effects of ageing, we further included age^2^ as a covariate.
